# Circulating tumor cells in cancer patients: developments and clinical applications for immunotherapy

**DOI:** 10.1186/s12943-020-1141-9

**Published:** 2020-01-24

**Authors:** Xiaoming Zhong, Hangtian Zhang, Ying Zhu, Yuqing Liang, Zhuolin Yuan, Jiachen Li, Jing Li, Xin Li, Yifan Jia, Tian He, Jiangyuan Zhu, Yu Sun, Wengting Jiang, Hui Zhang, Cheng Wang, Zunfu Ke

**Affiliations:** 1 Department of Pathology, The First Affiliated Hospital, Sun Yat-sen University, Guangzhou, Guangdong China; 2 School of Medicine, Sun Yat-sen University, Guangzhou, Guangdong China; 3 Department of Radiology, The First Affiliated Hospital, Sun Yat-sen University, Guangzhou, Guangdong China; 4 The Dietrich School of Arts and Sciences, University of Pittsburgh, Pittsburgh, Commonwealth of Pennsylvania, USA; 5 Precision Medicine Institute, The First Affiliated Hospital, Sun Yat-sen University, Guangzhou, Guangdong China; 6 Division of Nephrology, Department of medicine, The Fifth Hospital of Sun Yat-sen University, Zhuhai, 519000 Guangdong China

**Keywords:** Circulating tumor cells (CTCs), Isolation technologies, Prognosis, Immunotherapy, Immune mechanisms

## Abstract

Cancer metastasis is the leading cause of cancer-related death. Circulating tumor cells (CTCs) are shed into the bloodstream from either primary or metastatic tumors during an intermediate stage of metastasis. In recent years, immunotherapy has also become an important focus of cancer research. Thus, to study the relationship between CTCs and immunotherapy is extremely necessary and valuable to improve the treatment of cancer. In this review, based on the advancements of CTC isolation technologies, we mainly discuss the clinical applications of CTCs in cancer immunotherapy and the related immune mechanisms of CTC formation. In order to fully understand CTC formation, sufficiently and completely understood molecular mechanism based on the different immune cells is critical. This understanding is a promising avenue for the development of effective immunotherapeutic strategies targeting CTCs.

## Background

Cancer metastasis is the leading cause of cancer-related death and remains one of the prevailing challenges in cancer treatment. Most patients with metastatic disease are treated with systemic agents, which prolong survival and improve symptoms but are typically not curative, and patients are unable to achieve long-term survival [1]. In recent years, the prevailing view has become that metastatic disease is invariably widespread and incurable. However, with the emergence and success of cancer immunotherapy, notable exceptions exist, including subsets of patients with metastatic melanoma [2], non-small-cell lung cancer (NSCLC) [3], and renal cancer [4] treated with immunotherapy. In recent years, immunotherapy has become an important focus for cancer treatment, and it appears that immunotherapy combined with classical treatments, such as surgery, radiotherapy, and chemotherapy, can better improve patient survival rates [5]. Successful immunotherapeutic strategies require the identification of diagnostic, predictive, prognostic and therapeutic methods. Currently, the methods used in the clinic for guiding immunotherapies, such as tissue biopsy and imaging, are still not 100% accurate due to their limitations such as sensitivity and specificity. For instance, conventional tissue biopsy cannot always be routinely performed due to its invasive nature. Furthermore, the information acquired from a single biopsy only provides a limited snapshot of a tumor and often fails to reflect tumor heterogeneity. Therefore, it is critical to find a robust method for reflecting the overall biological characteristics of the tumor and assisting in making the optimal immunotherapy strategy [6].

A new diagnostic technique regarded as “liquid biopsy” has received considerable attention over the past several years [7, 8]. CTCs are one of the cornerstones of liquid biopsy and have indisputable advantages, as they are noninvasive, simple to administer, and more patient-friendly and would overcome the problem of tumor heterogeneity, allowing the progression of a tumor to be more easily followed by serial testing and helping to inform treatment decisions [9]. Recently, scientists have begun to explore the intrinsic relationships between immunotherapy and CTCs. The analysis of immune markers, heterogeneity and therapeutic targets from CTCs have shown promising application in immunotherapy. In this review, we systematically analyze the present isolation techniques for CTCs and then mainly investigate the clinical applications of CTCs in cancer immunotherapy and the related immune mechanisms of CTC formation.

## CTC isolation technologies

**Fig. 1:**
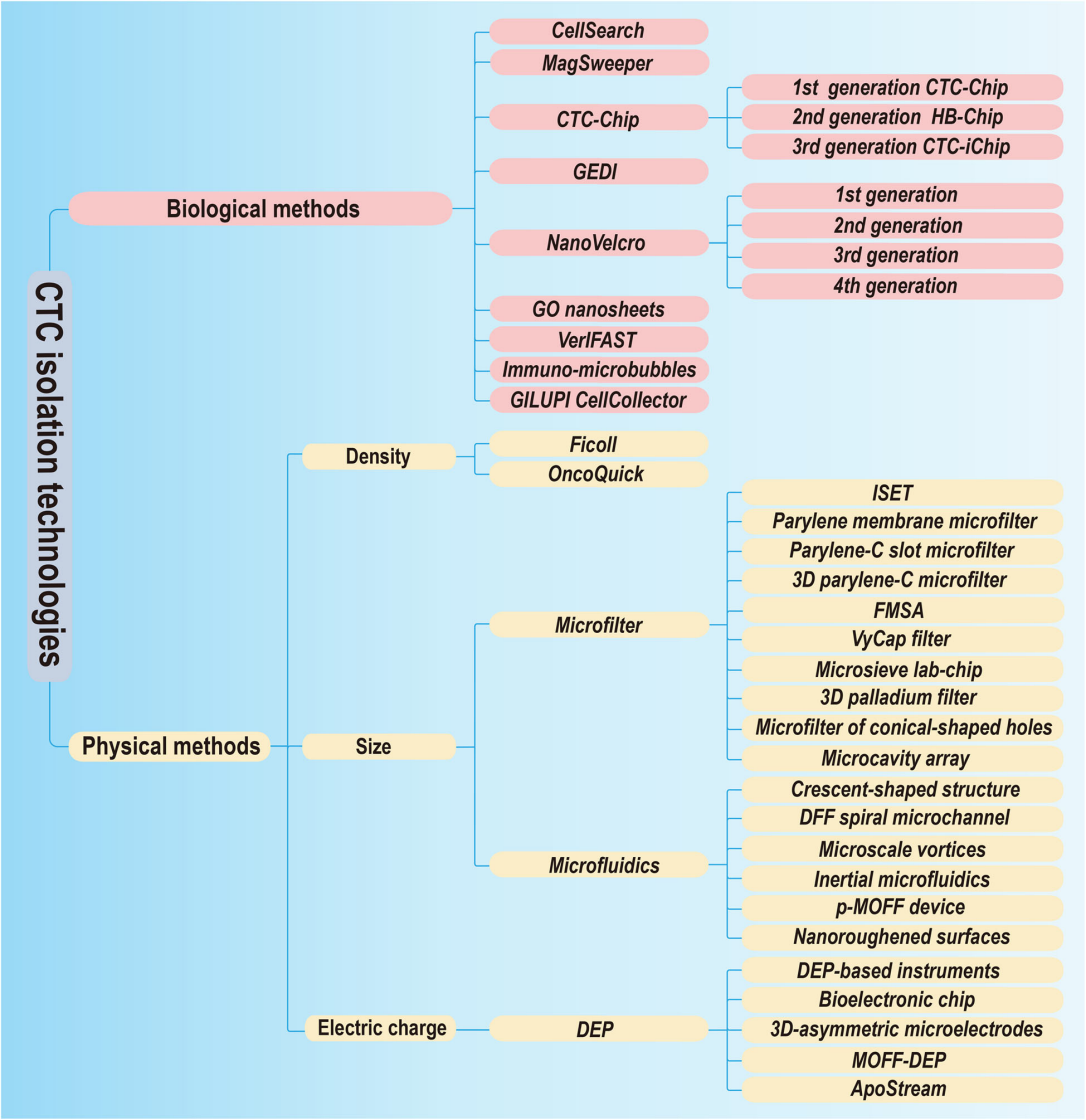
A mind map summarizing CTC isolation technologies. GEDI: geometrically enhanced differential immunocapture; GO: graphene oxide; *VerIFAST:* vertical *immiscible filtration assisted by surface tension*; ISET: isolation by size of epithelial tumor cells; FMSA: flexible micro spring array; *DFF: Dean Flow Fractionation;* p-MOFF: parallel multi-orifice flow fractionation; MOFF-DEP: multi-orifice flow fractionation and dielectrophoresis

CTCs are known as an important marker for auxiliary diagnosis, prognosis evaluation, treatment decision, etc. To further extend CTCs’ clinical application, it is necessary to develop specific and effective techniques to capture rare CTCs from peripheral blood. Here we generally classify all CTC isolation techniques into biological and physical methods according to their enrichment principles (Fig. 1).

### Biological isolation methods

Biological isolation methods are characterized by using specific surface markers, such as EpCAM. CellSearch is the gold standard for CTCs, capturing cells with specific EpCAM. The MagSweeper system introduces EpCAM-modified immunomagnetic beads, which are suitable for isolating circulating endothelial progenitor cells (CEpCs) with low to medium EpCAM expression. The three generations of the CTC-chip were developed to show increasingly higher isolation efficiency on CTCs, providing CTC samples with higher quality. The NanoVelcro chip is characterized by using specific antibody-modified nanomaterial substrate. One disadvantage of above methods is that they cannot effectively isolate CTCs with non-specific surface antigen expression. To overcome this defect, scientists are exploring new methods, even combining biological and physical isolation together, and achievements like CTC-iChip have been made (Additional file 1: Table S1).

### Physical isolation methods

Physical isolation methods are based on CTC physical properties such as size (microfilter), membrane charge (dielectrophoresis), and density (density gradient centrifugation), etc. The combination of physical properties with some specific platforms, such as microfluidics, also shows great potential in capturing CTCs. Most of these methods do not require specific surface markers on CTCs. These techniques are generally simple in principle but must depend advanced materials or assistive engineering technologies for better clinical application (Additional file 1: Table S1).

## The clinical applications of CTCs in immunotherapy

### Clinical prognosis prediction

The clinical prognostic value of CTCs has been being studied for years, but its predictive effect on immunotherapy is still insufficient. In this section, we will focus on the prognostic value of two aspects: the number and biological characteristics of CTCs (Additional file 2: Table S2). Mao et al. [10] found a significant decrease in the number of CTCs on days 7 and 30 after natural killer (NK) cell treatment in stage IV NSCLC, which may be related to the tumor shrinking. The tumor volume shrinks after NK cell treatment, which reduces the number of CTCs released from the lesion into the blood. Therefore, CTCs could be a useful biomarker for evaluating the efficacy of NK cell therapy. In another study of NK cell immunotherapy in hepatic carcinoma [11], a similar correlation was also observed. In addition, a study that aimed to investigate the safety and short-term efficacy of irreversible electroporation (IRE) combined with NK cell immunotherapy found that CTC number may reflect the efficacy of the combination therapy in unresectable primary liver cancer [12]. Currently, programmed cell death ligand 1 (PD-L1) expression is the most established predictive biomarker of the response to drugs that target the PD-L1/programmed cell death protein 1 (PD-1) axis [13–15]. To assess PD-L1 expression in tumors, tissue PD-L1 biopsy is a common method. However, this puts patients at risk of complications and delayed reports, and the limited sample may be inadequate to represent the overall tumor heterogeneity. PD-L1 expression on CTCs could offset the shortcoming of tissue PD-L1 biopsy. In patients treated with PD-1 inhibitor, pretreatment PD-L1+ CTCs are associated with their poor prognosis [16]. Based on PD-L1 expression on CTCs, after patients were treated with nivolumab for 6 months, they all obtained a clinical benefit in the group with PD-L1(−) CTCs, while they all experienced progressive disease in the PD-L1(+) CTC group [17]. In addition to NSCLC, CTCs are also predictors of worse outcomes in head and neck cancer (HNC). For an HNC cohort treated with nivolumab, CTC-positive patients had a shorter progression-free survival (PFS), and PD-L1-positive CTCs were found to be significantly associated with worse outcomes [18]. Specifically, in gastrointestinal tumors, high PD-L1 expression on CTCs at baseline might serve as a predictor to screen patients for PD-1/PD-L1 blockade therapies, and measuring the dynamic changes in CTCs could monitor the therapeutic response [19]. These reports indicate that a reduction in total CTC, PD-L1^posive^ CTC and PD-L1^high^ CTC counts may reflect a good response to PD-1 inhibitors (Additional file 2: Table S3). Additionally, the expression levels of MART-1, MAGE-A3 and PAX3 on CTCs have prognostic significance in patients with melanoma [20], and these proteins are highly expressed in melanoma tissues [21–25]. Multimarker RT-qPCR assay further demonstrated a significant association between the disease-free survival (DFS) and the expression levels of MART-1, MAGE-A3 and PAX3 [20, 21].

### Immunotherapeutic strategies targeting CTCs

#### Immune check point therapy

**Fig. 2:**
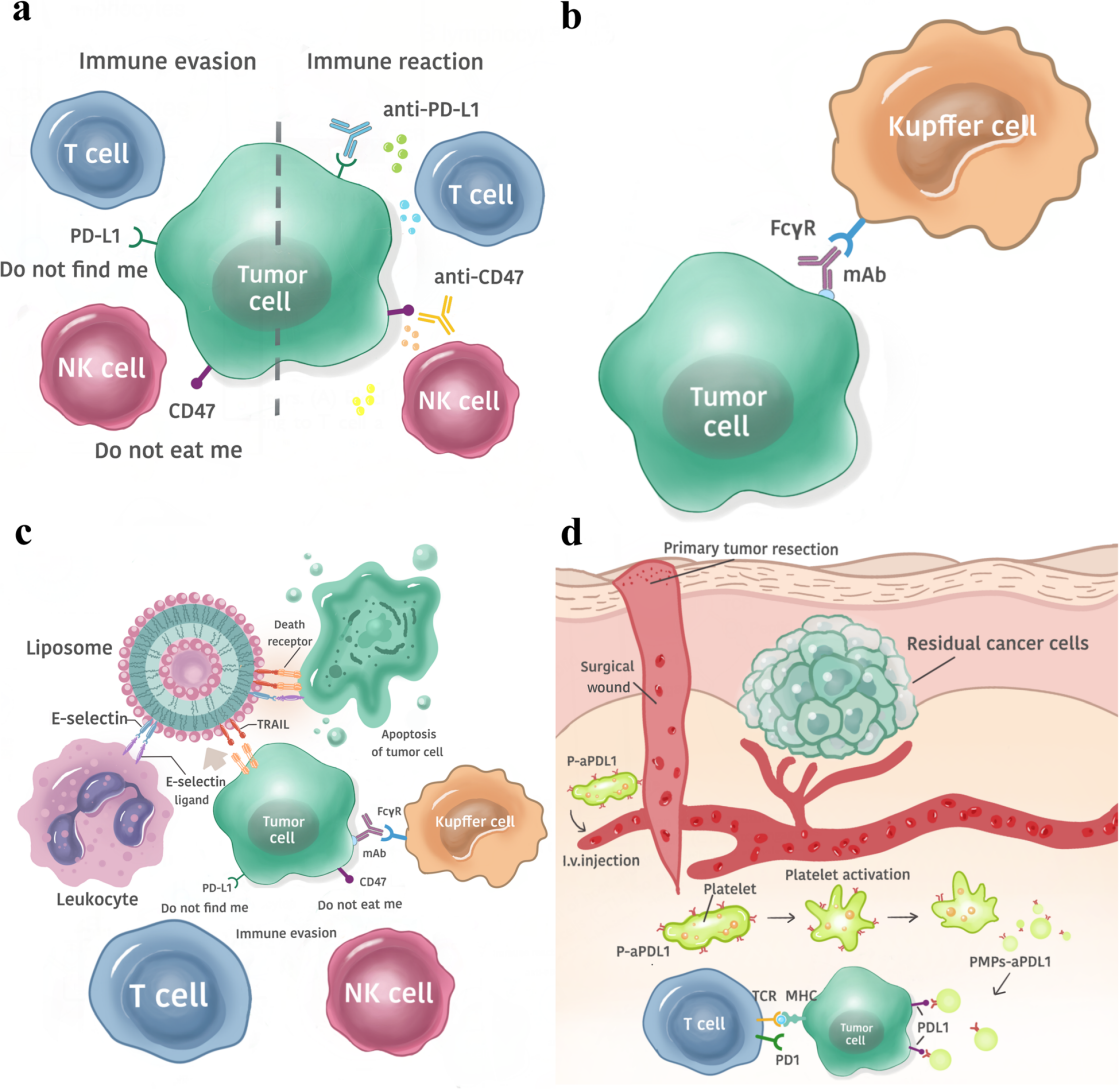
The four current immunotherapeutic strategies targeting circulating tumor cells. **a** Immune checkpoint therapy: The dual inhibition of both CD47 and PD-L1 inhibits immune evasion to promotes immune activation by T cells and NK cells. **b** Monoclonal antibody therapy: Depending on FcγRI and FcγRIV, monoclonal antibodies (mAbs) mediate CTC elimination by Kupffer cells. **c** “Unnatural killer cell” therapy: Leukocytes coated with E-selectin (ES)/tumor necrosis factor-related apoptosis inducing ligand (TRAIL) liposomes enhance the apoptotic effects of CTCs. **d** In vivo P-aPDL1 therapy: Conjugating anti-PDL1 (aPDL1) to the surface of platelets can facilitate the delivery of aPDL1 to target CTCs

Blocking immune checkpoints has been one of the focuses of antitumor immunotherapy in recent years (Fig. 2a) [26], and substantial progress has been made [27]. By blocking the immune checkpoint on CTCs, the immune system can be activated to eliminate CTCs in the blood circulation, which suggests a new way to reduce the recurrence and metastasis of malignant tumors. Using specific antibodies to simultaneously target two immune checkpoints, PD-L1 and CD47, was more effective than targeting PD-L1 or CD47 alone in inhibiting lung metastases [26].

A study proposed the concept of adaptive immune resistance [27], in which the tumor utilizes the natural physiology of PD-L1 induction to protect itself from an antitumor immune response. Therefore, the immune checkpoint PD-L1 can act as a “do not find me” signal on CTCs to escape the antitumor immune response. Blocking PD-L1 can enhance the activity of effector T cells and NK cells in the tumor microenvironment and may increase their production through indirect or direct effects on PD-1+ B cells. CD47 is also highly expressed on the surface of CTCs. CD47 can bind with signal regulatory protein α (SIRPα) on macrophages to transmit inhibitory signals and inhibit phagocytosis [28]. Therefore, CD47 can act as a “do not eat me” signal on CTCs. Blocking CD47 on CTCs can promote phagocytosis by macrophages. In addition, blocking CD47 can also promote macrophages or dendritic cells (DCs) to stimulate tumor-specific cytotoxic T cells, which can eventually clear CTCs [29].

Compared with using a single antibody, the combined blockade of CD47 and CD274 expression in tumors can cause the immune system to maintain a higher quality of T cells and NK cells in vivo and can prevent the immune escape of CTCs [26]. This immunotherapy with the dual blockade of immune checkpoints not only shows the interaction among CTCs, T cells, and NK cells in the immune microenvironment, but also provides a new direction for the targeted therapy based on immune checkpoint signal on CTC.

#### mAb therapy

In the decade from 2003 to 2013, the use of mAbs as therapeutic tools dramatically increased and became a mainstream strategy for cancer treatment (Fig. 2b) [30], but how mAbs specifically mediate tumor cell elimination and the effects involved in the process are still unclear. Until 2013, based on in vitro live cell imaging and in vivo microscopy of the mouse liver, the researchers proposed the mode of action of mAbs, which for the first time, directly demonstrated that mAb therapy induced the macrophage phagocytosis of CTCs and that this effect was dependent on FcγRI and FcγRIV [30]. This conclusion was consistent with that of their earlier studies, which demonstrated that FcγRI and FcγRIV were required to prevent liver metastasis after mAb treatment [31].

In the mouse model system, the B16F10 cell line, is the only homologous mouse solid tumor cell line [32] that can be used to obtain specific mAbs. Mice were vaccinated with B16F10 cells and were treated with a vector or TA99 mAb. In vivo imaging in the liver of mice treated with the vector showed that Kupffer cells were able to interact with a small portion of tumor cells without causing the elimination of tumor cells. However, Kupffer cells in the liver of mice treated with the TA99 mAb were able to rapidly recognize and phagocytose tumor cells. Although there was no difference in the number of tumor cells that contacted Kupffer cells in the liver of mice treated with the vector or the TA99 mAb, the number of phagocytosed tumor cells significantly increased after treatment with the TA99 mAb. Repeated experiments with isotype mAbs were carried out to further confirm the conclusion and to rule out the possibility of nonspecific phagocytosis due to the injection of mAbs [32]. To investigate whether other non-Kupfer cell-dependent killing occurred, clodronate liposomes were used to deplete Kupffer cells [33] before the injection of tumor cells and mAbs. When the cells were depleted, treatment with the TA99 mAb was ineffective.

For patients with primary colorectal cancer, tumor resection creates a permissive environment for tumor cells to adhere to the liver and increases the risk of metastasis, while Kupffer cells are the first defense line for tumor cells to enter into the liver. Kupffer cells are able to sample small number of tumor cells without mAbs [34] but do not block tumor cells very effectively. In contrast, after mAb treatment, Kupffer cells effectively phagocytosed intact tumor cells, thereby preventing liver metastasis.

#### “Unnatural killer cell” therapy

The use of TRAIL- and ES-coated white blood cells (WBCs) to reduce CTCs is suggested to be very effective (Fig. 2c), both in vitro in human blood and in vivo in mice [35]. To form a distant metastasis, CTCs have to cross vascular endothelial cells, similar to WBCs. Therefore, CTCs possess the characteristics that overlap with WBCs, such as surface molecules, which are involved in adhesion to endothelial cells. Further, CTCs possess the activity similar to the inflammatory infiltration and lymphocyte homing processes and thereby penetrate endothelial cells to form tiny metastases [36–41]. In many tumor-derived CTCs, surface-expressed glycosylated ligands are capable of recognizing and binding to ESs expressed on endothelial cells [42]. In a liposome (Fig. 2c) containing ES and TRAIL, the interaction between ES on tumor cells and the death receptor TRAIL on COLO 205 cells and PC-3 cells induced autophagy in tumor cells. However, in the bloodstream, the large number of blood cells and the small number of tumor cells [43] make it difficult for the liposomes to effectively and frequently contact CTCs. In the blood stream, red blood cells occupy the center of the laminar flow, while CTCs and WBCs are located in the outer layer of the flow, which causes CTCs to contact WBCs more frequently [35, 44]. Furthermore, the leukocyte surface also contains an ES receptor. Thus, WBCs carrying ES and TRAIL liposomes can allow TRAIL to more effectively contact CTCs, promoting CTC phagocytosis and controlling hematogenous metastasis by reducing the number of CTCs. Although this method did effectively inhibit tumor cells in the experimental stage, it remains to be seen whether it can reduce the formation of metastases [35].

#### In vivo P-aPD-L1 therapy

Platelets play a critical role in tumor thrombus formation and tumor metastasis. Tumor cells induce platelet activation and aggregation in the blood circulation (Fig. 2d) [45]. At the same time, tumor cells and platelets form tumor thrombi by releasing thrombin-activated fibrinogen [46].

Platelets can capture CTCs in a variety of ways, such as via P-selection, via the indirect capture of tumor cells through the coagulation system, and via the capture of tumor cells through the immune complement pathway [47]. Additionally, platelets can promote tumor metastasis by aggregating with CTCs, thus helping CTCs avoid immune attack and migrate to new tissues, during which the binding between P-selectin and the CD44 receptor plays a key role [46, 48]. CTCs can interact with activated platelets and leukocytes and can form aggregates that attach to endothelial cells, which contribute to metastasis [49].

PD-1 is a coinhibitory receptor expressed on the surface of antigen-stimulated T cells. PD-L1 is a protein that is encoded by the CD274 gene [50]. PD-1/PD-L1 inhibitors can block the PD-1/PD-L1 pathway and can promote T cells from attacking tumor cells [51]. Based on the interaction between platelets and cancer cells, a platelet stimulating drug delivery system has been developed [52]. One technique involves binding aPD-L1 to the platelet surface to form aPD-L1-conjugated platelets (P–aPD-L1). This binding is highly stable without causing any significant platelet damage [45]. When vascular endothelial cells are damaged, receptors on the surface of platelets bind to their corresponding ligands. Platelets adhere to the injury site and become activated; then, their contents are released into the extracellular environment in the form of particles, leading to the recruitment and activation of other immune cells as well as to T cell migration and monocyte differentiation into DCs [53]. At the same time, pseudopods form around the activated platelets, and the serosa fall off to form platelet-derived microparticles (PMPs) [54]. Conjugated aPDL1 is also present on the PMP membrane. PMPs can promote the targeted binding of conjugated aPDL1 to CTCs and antigen presenting cells (APCs) in peripheral blood, thus blocking the expression of PD-L1 on tumor and APCs, reducing local tumor recurrence and inhibiting tumor metastasis.

When P-aPDL1 was injected into mice with partially resected primary melanoma (B16F10) or into a triple-negative breast cancer (TNBC) tumor model (4 T1 carcinoma), aPDL1 was effectively released through platelet-derived particles during platelet activation. aPDL1 significantly reduced the risk of cancer recurrence and metastasis and prolonged the overall survival time of mice after the operation. Additionally, P–aPDL1 therapy has a stronger anticancer effect than free-aPDL1 treatment. One of the reasons is that the local concentrations of antibodies increase around cancer cells. Another reason is that platelet activation not only induces the release of conjugated aPDL1, but also recruits many other immune cells into the tumor microenvironment. Upon blocking PD-L1, these immune cells can induce a strong anticancer immune response [45].

In regard to using the interaction between platelets and CTCs for immunotherapy, therapeutic drugs other than aPDL1 can be selected to bind to the platelet surface. Chen et al. coated PM-NV composites containing acid-sensitive cross-linking agents in platelet membranes and modified platelet membranes with TRAIL. Platelets can target PM-NV composites loaded with drugs to tumor cells, and then the drugs are released and inhibit the development of tumors [52].

#### Interaction between tumor cells and immune cells or cellular components

The immune system and tumor microenvironment play a decisive role in tumor progression. A novel 4D lung model (see later in the article for a description of the model) was developed to better understand tumor progression and the interaction between tumor and immune cells or cellular components [55].

First, CTCs from the 4D lung cancer model were injected into immune competent mice and nu/nu mice, respectively. In the immune competent mice, tumor cell lines did not form metastatic lesions, while in the nu/nu mice, metastases formed. This highlights the important role of immune cells in inhibiting the formation of metastatic lesions. Second, a cellular 4D model in which all of the cells in the lung were preserved was used to model the in vivo phenomenon. The naïve immune cells and activated immune cells were added to the model, which was seeded with tumor cell lines; while the activated cell line inhibited metastasis, and the naïve cell line did not. This further emphasizes the importance of activated immune cells in inhibiting the formation of metastatic lesions. Third, genes related to immune regulation and metastasis were compared between nonmetastatic cell lines and metastatic cell lines in the model with activated immune cells. The results showed that the expression of PD-L1 in the metastatic cell line was significantly higher than that in the nonmetastatic cell lines in the model. In general, activated immune cells impact the activity of CTCs that have decreased PD-L1 expression, resulting in the inhibition of metastatic lesion formation [55]. This study suggests a possible immunotherapy approach to inhibit tumor metastasis by reducing the activity of CTCs. Namely, the expression of PD-L1 on CTCs could be inhibited or the effect of PD-L1 on CTCs could be blocked.

#### Cellular models for studying immunotherapy targeting CTCs

##### 4 T1 cell line

4 T1 cells are 6-thioguanine-resistant cells selected from the 410.4 tumor cell line without mutagenesis. When 4 T1 cells are injected into BALB/c mice, a primary tumor lesion can form at the injection site, and 4 T1 cells can spontaneously form highly metastatic tumors that can metastasize to the lungs, liver, lymph nodes and brain. The growth and metastatic characteristics of 4 T1 cells in BALB/c mice are very similar to those in human breast cancer, so tumors from 4 T1 cells can be used as an animal model of human breast cancer. Even small clusters of metastatic cells (as few as one) in distal organs could also be detected. Therefore, the 4 T1 cell line can be used to study the metastasis of CTCs at the distal site. To evaluate whether synergistically blocking CD47 and CD274 on cancer cells was effective against CTCs in the lungs, a well-established CTC 4 T1 model was employed [26].

##### B16 cell line

B16 cells are a useful model for studying metastasis and solid tumor formation and one of the first effective murine tools for metastasis research. B16 cells originate in the melanogenic epithelia of mice and are easy to track in vivo posttransplantation. Their fidelity of metastasis from skin to the lung, liver, and spleen make them a useful and predictable tool to study metastatic pathways. B16 cells are also used as a preclinical model to study immunotherapy [56]. Among B16 cells, the B16F10 cell line has the strongest ability to metastasize and undergo erosion. B16F10 CTCs could be detected in the blood circulation on the fourth day after the subcutaneous inoculation of tumor cells [57].

##### Cellular and acellular 4D lung cancer model

The ex vivo cellular 4D model was created by harvesting the heart-lung block from Sprague-Dawley rats, while the acellular 4D model was developed by removing native lung cells, which leaves behind the native extracellular matrix [55]. The native matrix components provide an intact structure with the vasculature, bronchi and alveoli. In the experiment, tumor cells (344SQ or 393P) were placed in the left trachea, traveled to the left lung and formed a primary tumor. Later, the acellular and cellular lungs were connected to the right main bronchus to form a metastasis model in which the CTCs break away from the primary tumor, intravasate into the vasculature, travel to the contralateral lung, extravasate and form metastatic lesions. This model allows the isolation of tumor cells at different phases of tumor progression, namely, at the primary tumor site, in the circulation, and from metastatic lesions, which aids in the study of the mechanism of CTC metastasis. By adding immune cells to the model, the mechanism of immune cell interactions with tumor cells and the impact of this interaction on metastasis can also be studied, providing a new direction for tumor immunotherapy [55].

## CTC formation: relevant immune mechanisms

The process of CTC formation and metastasis involves several main steps: cancer cell release, immune escape, and adhesion to and exudation from blood vessels to form distant metastases. In these processes, interactions between CTCs and immune system play an important role. Although thousands of tumor cells enter the blood from the primary tumor per day on average, the number of CTCs that can be actually measured is often very small. This is because a large number of tumor cells are more likely to be attacked by immune cells due to the loss of the protection from the original immunosuppressive microenvironment after their release.

**Fig. 3:**
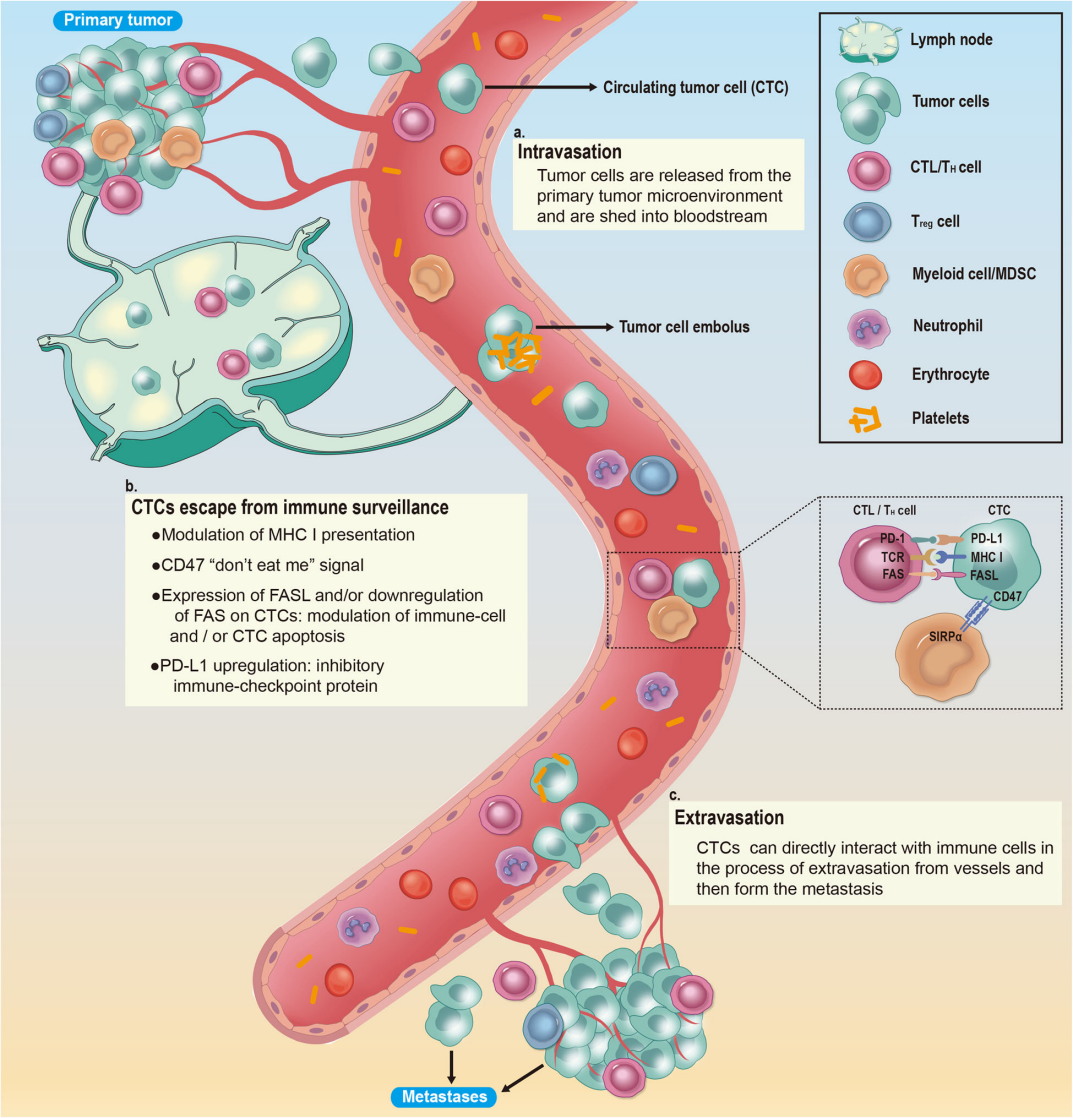
The metastatic cascade: The main steps of tumor spread. **a**. Intravasation: Tumor cells are first released from the primary tumor microenvironment, then traverse the interstitial connective tissue, and ultimately gain access to the circulation by penetrating the vascular basement membrane. **b**. CTCs escape from immune surveillance in the circulation: CTCs encounter immune cells through direct cell–cell interactions and are subject to immune-mediated elimination. Escape mechanisms involving the expression of CD47, PD-L1 and FASL, as well as alterations in MHC molecules, promote the survival of CTCs in the circulation. **c**. Extravasation: In the process of extravasating to secondary locations, CTCs can directly interact with immune cells, supporting the formation of metastases

The first process is the release of tumor cells, which is mainly associated with tumor angiogenesis, the alteration of the extracellular microenvironment and the loss of cell adhesion molecules. The major immune components in this process include tumor-associated macrophages (TAMs), myeloid-derived suppressor cells (MDSCs), neutrophils, and platelets. For example, MDSCs secrete proinflammatory factors and endothelial growth factors to induce tumor angiogenesis [58]. In addition, MDSCs secrete IL-6, TGF-β, EGF and HFG to promote epithelial-mesenchymal transition (EMT) in tumor cells [59, 60]. Platelets release growth factors such as PDGF, EGF and VEGF to induce tumor angiogenesis and increase the permeability of blood vessels by releasing MMPs, 5-hydroxytryptamine and histamine. MDSCs, TAMs, and neutrophils can produce various proteases, such as matrix metalloproteinase 9 (MMP-9), to promote matrix digestion and remodeling and promote tumor cell migration and extravasation into blood vessels by secreting cytokines [61, 62]. The paracrine loop of TAMs and tumor cells also plays an important role in mediating tumor invasion and metastasis [63]. Furthermore, platelets and neutrophils can promote the adhesion of CTCs to endothelial cells [64, 65]. Neutrophils can also capture and adhere to CTCs through neutrophil extracellular traps (NETs) [66]. Studies have discovered that the development and metastasis of advanced melanoma is correlated with MDSCs, Treg cells and the levels of IL-1β, IFNγ, and CXCL10 in peripheral blood [67]. With regard to the immune escape of CTCs, the more detailed mechanism will be described below based on the different immune cells (Fig. 3).

### Dendritic cells (DCs)

Clinical studies have demonstrated that there are significant correlations between the number of CTCs and the number of DCs [68]. DCs can become tumor-associated DCs with an impaired self-function under the influence of the tumor environment, which can affect the recognition and killing functions of cytotoxic T lymphocytes (CTLs), NK cells and other cells [68].

### Cytotoxic T lymphocytes (CTLs)

The T cell receptors (TCRs) on the surface of CTLs can specifically recognize tumor-associated antigens presented by MHC-I molecules on the surface of tumor cells. To escape this killing effect, MHC-I molecules are expressed at lower or even undetectable levels in many tumor cells [69]. In addition, the expression of other molecules on the surface of tumor cells can also influence this mutual recognition. The overexpression of Cytokeratin 8 (CK8), together with its heterodimeric partners CK18 and CK19, on the surface of tumor cells has been demonstrated to inhibit MHC I interactions with TCRs on CD8+ CTLs [70, 71]. In addition to preventing specific T cell recognition, tumor cells also kill T cells by upregulating the expression of FASL on their surface while downregulating the expression of FAS, which reduces the threshold for apoptosis in CTLs, to achieve immune escape [72]. This mechanism mainly leads to the apoptosis of some CD8+ T cells [73]. Some other experiments suggest that CTCs may escape immune attack by secreting soluble FASL [74–76]**.** Blocking immune checkpoints is another important immune escape mechanism, and PD-1 and PD-L1 are the most prominent examples. PD-L1 can be expressed by tumor cells and can transmit inhibitory signals after binding to PD-1 on T cells, thereby limiting immune effector functions [27] CTL associated antigen 4 (CTLA 4), related B7 family members and galectin 9 are also possible targets for immune escape mechanisms [77]. Several studies have demonstrated that when HLA-G or a nonclassical MHC I are highly expressed on the surface of tumor cells, the killing effect of T cells and NK cells can be inhibited [78–81]. HLA-G inhibits the process in which immune cells destroy tumor cells by binding to a multitude of receptors, such as KIRs, CD8, and leukocyte immunoglobulin like receptor sub family B member 1 (LIR 1), which are expressed on the surface of immune cells. The secretion of soluble HLA G (sHLA G), a molecule that results from alternative splicing within cancer cells, is also a mechanism of immune escape [82].

### NK cells

With regard to the immune escape mechanisms of NK cells, on the one hand, tumor cells can undergo changes that make it difficult for NK cells to recognize and kill them. On the other hand, tumor cells actively secrete some substances that inhibit NK cell activity [83]. NK cells mainly identify tumor cells and initiate the killing process by recognizing MICA/MICB on tumor cells through the NKG2D receptor. Therefore, tumor cells mainly downregulate the expression of MICA/MICB on the surface while upregulating the expression of hypoxia inducible factor 1α (HIF 1α) to increase the cell surface expression of disintegrin and metalloproteinase containing domain protein 10 (ADAM10), which can cleave surface MICA/MICB [84, 85]. Moreover, in glioblastoma, tumor cells induced NK cell activation via the secretion of lactate dehydrogenase 5 (LDH5), resulting in the decreased expression of surface NKG2D receptors [86]. Notably, while the inhibition of NKG2D receptor activation is a way that tumors escape NK cell killing in many studies, there are still a few experiments where the results appear to contradict to our current understanding. For example, a soluble MHC I related NKG2D ligand (Mult1) stimulated NK-mediated antitumor responses in an experiment [87]. Additionally, CTCs have been shown to inhibit the activity of NK cells by causing platelet to aggregate and interact with NK cells [88, 89].

### Macrophages

Macrophages play a major role in removing CTCs from the blood. In particular, resident macrophages in the liver show a strong ability to clear CTCs. Studies showed that some CTCs can upregulate the expression of CD47 on their surface, which is identified by SIRPα (also known as macrophage fusion receptor) on the surface of macrophages and DCs, then transmitting the ‘do not eat me’ signal and inhibiting the clearance of tumor cells [28]. Although numerous studies demonstrated the consequences of CD47 expression in relation to immune escape [90, 91] and indicated that it might be a part of a potential metastasis initiator signature, up to now, this mechanism has not been clear enough [49].

### Platelets

Platelets can rapidly adhere to CTCs and can transfer platelet-specific MHC class I to tumor cells, thereby escaping recognition and killing by NK cells [69]. In response to DCs, the most potent APCs in tumor immunity, VEGF is released from platelets and can inhibit the differentiation and development of DCs. In vitro platelets can prevent the differentiation of hematopoietic precursors into DCs [92, 93]. TGFβ released from platelets can also inhibit immune function in various ways, such as inhibiting the infiltration, proliferation, differentiation, and activation of immune cells in tumors, inducing low or no expression of HLA-class II molecules, etc., allowing tumor cells to escape immune surveillance [94].

## Conclusion

Along with the development of CTC isolation technologies and the progress of tumor immune research, CTCs have begun to be considered an immunotherapeutic target, and adopting immunotherapeutic strategies to reduce or even eliminate CTCs may be a new and feasible way to inhibit tumor metastasis or recurrence. However, due to insufficiently and incompletely understood molecular mechanisms, immunotherapeutic strategies targeting CTCs are not currently fully developed. We look forward to more further research on the relationships between CTC formation and immune escape.

## Supplementary information

**Additional file 1 : Table S1.** Biological and physical isolation techniques of CTC.

**Additional file 2 : Table S2.** Current studies on the prognostic value of CTCs in immunotherapeutic strategies. **Table S3.** PD-L1 status in CTCs of patients before and after the initiation of IBI308 therapy.

## Abbreviations

APC: Antigen presenting cells
aPDL1: Anti-PDL1
B7-H1: B7 homolog1
CK8: Cytokeratin 8
CTCs: Circulating tumor cells
CTL: Cytotoxic T lymphocyte
*DFF*: *Dean Flow Fractionation*
DFS: Disease free surviving
FASL: FAS ligand
FMSA: Flexible micro spring array
GEDI: Geometrically enhanced differential immunocapture
GO: Graphene oxide
HNC: Head and neck cancer
IRE: Irreversible electroporation
ISET: Isolation by size of epithelial tumor cells
MDSCs: Myeloid-derived suppressor cells
MHC I: Major histocompatibility complex class I
MMP-9: matrix metalloproteinase 9
MOFF-DEP: Multi-orifice flow fractionation and dielectrophoresis
NETs: Neutrophils extracellular traps
NK: Natural killer
NSCLC: Non-small-cell lung cancer
P–aPDL1: aPDL1-conjugated platelets
PD: Progressive disease
PD1: Programmed cell death protein 1
PD-L1: Programmed cell death ligand 1
PFS: Progression-free survival
p-MOFF: Parallel multi-orifice flow fractionation
PMPs: Platelet-derived microparticles
PR: Partial response
SD: Stable disease
SIRPα: Signal regulatory protein α
TAMs: Tumor-associated macrophages
TCR: T cell receptor
TH: T Helper
TNBC: Triple negative breast cancer
Treg: T-Regulatory
*VerIFAST*: Vertical *immiscible filtration assisted by surface tension*
WBC: White blood cells
