# Supplementary material for: Circulating tumor cells in cancer patients: developments and clinical applications for immunotherapy
Source: Mol Cancer. 2020 Jan 24;19:15. doi: 10.1186/s12943-020-1141-9 (PMC6982393; doi:10.1186/s12943-020-1141-9)
Supplement: Supplementary file 1 — Additional file 1 : Table S1. Biological and physical isolation techniques of CTC. [file 12943_2020_1141_MOESM1_ESM.docx]

**Table S1.** Biological and physical isolation techniques of CTC

| Biological methods | CellSearch | none | | [1] |
| --- | --- | --- | --- | --- |
|  | MagSweeper | none | | [2] |
|  | CTC-Chip | 1^st^ generation CTC-Chip | | [3] |
|  |  | 2^nd^ generation HB-Chip | | [4] |
|  |  | 3^rd^ generation CTC-iChip | | [5] |
|  | GEDI | none | | [6] |
|  | NanoVelcro | 1^st^ generation | | [7] |
|  |  | 2^nd^ generation | | [8] |
|  |  | 3^rd^ generation | | [9] |
|  |  | 4^th^ generation | | [10] |
|  | GO nanosheets | none | | [11] |
|  | VerIFAST | none | | [12] |
|  | Immuno-microbubbles | none | | [13] |
|  | GILUPI CellCollector | none | | [14] |
| Physical methods | Density | Ficoll |  | [15] |
|  |  | OncoQuick |  | [15] |
|  | Size | Microfilter | ISET | [16] |
|  |  |  | Parylene membrane microfilter | [17] |
|  |  |  | Parylene-C slot microfilter | [18] |
|  |  |  | 3D parylene-C microfilter | [19] |
|  |  |  | FMSA | [20] |
|  |  |  | VyCap filter | [21] |
|  |  |  | Microsieve lab-chip | [22] |
|  |  |  | 3D palladium filter | [23] |
|  |  |  | Microfilter of conical-shaped holes | [24] |
|  |  |  | Microcavity array | [25, 26] |
|  |  | Microfluidics | Crescent-shaped structure | [27] |
|  |  |  | DFF spiral microchannel | [28] |
|  |  |  | Microscale vortices | [29] |
|  |  |  | Inertial microfluidics | [30, 31] |
|  |  |  | p-MOFF device | [32] |
|  |  |  | Nanoroughened surfaces | [33] |
|  | Electric charge | DEP | DEP-based instruments | [34, 35] |
|  |  |  | Bioelectronic chip | [36] |
|  |  |  | 3D-asymmetric microelectrodes | [37] |
|  |  |  | MOFF-DEP | [38] |
|  |  |  | ApoStream | [39] |

References

1. Allard WJ, Matera J, Miller MC, Repollet M, Connelly MC, Rao C, et al. Tumor cells circulate in the peripheral blood of all major carcinomas but not in healthy subjects or patients with nonmalignant diseases**.** Clin Cancer Res. 2004; 10(20):6897-904.

2. Talasaz AH, Powell AA, Huber DE, Berbee JG, Roh KH, Yu W, et al. Isolating highly enriched populations of circulating epithelial cells and other rare cells from blood using a magnetic sweeper device. Proc Natl Acad Sci U S A. 2009; 106(10):3970-75.

3. Nagrath S, Sequist LV, Maheswaran S, Bell DW, Irimia D, Ulkus L, et al. Isolation of rare circulating tumour cells in cancer patients by microchip technology. Nature. 2007; 450(7173):1235-9.

4. Stott SL, Hsu CH, Tsukrov DI, Yu M, Miyamoto DT, Waltman BA, et al. Isolation of circulating tumor cells using a microvortex-generating herringbone-chip. Proc Natl Acad Sci U S A. 2010; 107(43):18392-7.

5. Ozkumur E, Shah AM, Ciciliano JC, Emmink BL, Miyamoto DT, Brachtel E, et al. Inertial focusing for tumor antigen-dependent and -independent sorting of rare circulating tumor cells. Sci Transl Med. 2013; 5(179):179ra147.

6. Kirby BJ, Jodari M, Loftus MS, Gakhar G, Pratt ED, Chanel-Vos C, et al. Functional characterization of circulating tumor cells with a prostate-cancer-specific microfluidic device. PLoS One. 2012; 7(4):e35976.

7. Wang S, Liu K, Liu J, Yu ZT, Xu X, Zhao L, et al. Highly efficient capture of circulating tumor cells by using nanostructured silicon substrates with integrated chaotic micromixers. Angew Chem Int Ed Engl. 2011; 50(13):3084-8.

8. Zhao L, Lu YT, Li F, Wu K, Hou S, Yu J, et al. High-purity prostate circulating tumor cell isolation by a polymer nanofiber-embedded microchip for whole exome sequencing. Adv Mater. 2013; 25(21):2897-902.

9. Hou S, Zhao H, Zhao L, Shen Q, Wei KS, Suh DY, et al. Capture and stimulated release of circulating tumor cells on polymer-grafted silicon nanostructures. Adv Mater. 2013; 25(11):1547-51.

10. Jan YJ, Chen JF, Zhu Y, Lu YT, Chen SH, Chung H, et al. NanoVelcro rare-cell assays for detection and characterization of circulating tumor cells. Adv Drug Deliv Rev. 2018; 125:78-93.

11. Yoon HJ, Kim TH, Zhang Z, Azizi E, Pham TM, Paoletti C, et al. Sensitive capture of circulating tumour cells by functionalized graphene oxide nanosheets. Nat Nanotechnol. 2013; 8(10):735-41.

12. Casavant BP, Guckenberger DJ, Berry SM, Tokar JT, Lang JM, Beebe DJ. The VerIFAST: an integrated method for cell isolation and extracellular/intracellular staining. Lab Chip. 2013;, 13(3):391-6.

13. Shi G, Cui W, Benchimol M, Liu YT, Mattrey RF, Mukthavaram R, et al. Isolation of rare tumor cells from blood cells with buoyant immuno-microbubbles. PLoS One. 2013; 8(3):e58017.

14. Saucedo-Zeni N, Mewes S, Niestroj R, Gasiorowski L, Murawa D, Nowaczyk P, et al. A novel method for the in vivo isolation of circulating tumor cells from peripheral blood of cancer patients using a functionalized and structured medical wire. Int J Oncol. 2012; 41(4):1241-50.

15. Rosenberg R, Gertler R, Friederichs J, Fuehrer K, Dahm M, Phelps R, et al. Comparison of two density gradient centrifugation systems for the enrichment of disseminated tumor cells in blood. Cytometry. 2002; 49(4):150-8.

16. Vona G, Sabile A, Louha M, Sitruk V, Romana S, Schutze K, et al. Isolation by size of epithelial tumor cells: a new method for the immunomorphological and molecular characterization of circulatingtumor cells. Am J Pathol. 2000; 156(1):57-63.

17. Zheng S, Lin H, Liu JQ, Balic M, Datar R, Cote RJ, et al. Membrane microfilter device for selective capture, electrolysis and genomic analysis of human circulating tumor cells. J Chromatogr A. 2007; 1162(2):154-61.

18. Xu T, Lu B, Tai YC, Goldkorn A. A cancer detection platform which measures telomerase activity from live circulating tumor cells captured on a microfilter. Cancer Res. 2010; 70(16):6420-6.

19. Zheng S, Lin HK, Lu B, Williams A, Datar R, Cote RJ, et al. 3D microfilter device for viable circulating tumor cell (CTC) enrichment from blood. Biomed Microdevices. 2011; 13(1):203-13.

20. Harouaka RA, Zhou MD, Yeh YT, Khan WJ, Das A, Liu X, et al. Flexible micro spring array device for high-throughput enrichment of viable circulating tumor cells. Clin Chem. 2014; 60(2):323-33.

21. Coumans FA, van Dalum G, Beck M, Terstappen LW. Filter characteristics influencing circulating tumor cell enrichment from whole blood. PLoS One. 2013; 8(4):e61770.

22. Lim LS, Hu M, Huang MC, Cheong WC, Gan AT, Looi XL, et al. Microsieve lab-chip device for rapid enumeration and fluorescence in situ hybridization of circulating tumor cells. Lab Chip. 2012; 12(21):4388-96.

23. Yusa A, Toneri M, Masuda T, Ito S, Yamamoto S, Okochi M, et al. Development of a new rapid isolation device for circulating tumor cells (CTCs) using 3D palladium filter and its application for genetic analysis. PLoS One. 2014; 9(2):e88821.

24. Tang Y, Shi J, Li S, Wang L, Cayre YE, Chen Y. Microfluidic device with integrated microfilter of conical-shaped holes for high efficiency and high purity capture of circulating tumor cells. Sci Rep. 2014; 4:6052.

25. Hosokawa M, Hayata T, Fukuda Y, Arakaki A, Yoshino T, Tanaka T, et al. Size-selective microcavity array for rapid and efficient detection of circulating tumor cells. Anal Chem. 2010; 82(15):6629-35.

26. Hosokawa M, Yoshikawa T, Negishi R, Yoshino T, Koh Y, Kenmotsu H, et al. Microcavity array system for size-based enrichment of circulating tumor cells from the blood of patients with small-cell lung cancer. Anal Chem. 2013; 85(12):5692-8.

27. Tan SJ, Yobas L, Lee GY, Ong CN, Lim CT. Microdevice for the isolation and enumeration of cancer cells from blood. Biomed Microdevices. 2009; 11(4):883-92.

28. Hou HW, Warkiani ME, Khoo BL, Li ZR, Soo RA, Tan DS, et al. Isolation and retrieval of circulating tumor cells using centrifugal forces. Sci Rep. 2013; 3:1259.

29. Hur SC, Mach AJ, Di Carlo D.: High-throughput size-based rare cell enrichment using microscale vortices. Biomicrofluidics. 2011; 5(2):22206.

30. Hur SC, Henderson-MacLennan NK, McCabe ER, Di Carlo D. Deformability-based cell classification and enrichment using inertial microfluidics. Lab Chip. 2011; 11(15):912-20.

31. Bhagat AA, Hou HW, Li LD, Lim CT, Han J. Pinched flow coupled shear-modulated inertial microfluidics for high-throughput rare blood cell separation. Lab Chip. 2011; 11(11):1870-8.

32. Hyun KA, Kwon K, Han H, Kim SI, Jung HI. Microfluidic flow fractionation device for label-free isolation of circulating tumor cells (CTCs) from breast cancer patients. Biosens Bioelectron. 2013; 40(1):206-12.

33. Chen W, Weng S, Zhang F, Allen S, Li X, Bao L, et al. Nanoroughened surfaces for efficient capture of circulating tumor cells without using capture antibodies. ACS Nano. 2013; 7(1):566-75.

34. Becker FF, Wang XB, Huang Y, Pethig R, Vykoukal J, Gascoyne PR. Separation of human breast cancer cells from blood by differential dielectric affinity. Proc Natl Acad Sci U S A. 1995; 92(3):860-4.

35. Gascoyne PR, Shim S. Isolation of circulating tumor cells by dielectrophoresis. Cancers (Basel). 2014; 6(1):545-79.

36. Cheng J, Sheldon EL, Wu L, Heller MJ, O'Connell JP. Isolation of cultured cervical carcinoma cells mixed with peripheral blood cells on a bioelectronic chip. Anal Chem. 1998; 70(11):2321-6.

37. Park J, Kim B, Choi SK, Hong S, Lee SH, Lee KI. An efficient cell separation system using 3D-asymmetric microelectrodes. Lab Chip. 2005; 5(11):1264-70.

38. Moon HS, Kwon K, Kim SI, Han H, Sohn J, Lee S, et al. Continuous separation of breast cancer cells from blood samples using multi-orifice flow fractionation (MOFF) and dielectrophoresis (DEP). Lab Chip. 2011; 11(6):1118-25.

39. Gupta V, Jafferji I, Garza M, Melnikova VO, Hasegawa DK, Pethig R, et al. ApoStream^TM^, a new dielectrophoretic device for antibody independent isolation and recovery of viable cancer cells from blood. Biomicrofluidics. 2012; 6(2):24133.
