# Supplementary material for: Circulating tumor cells in cancer patients: developments and clinical applications for immunotherapy
Source: Mol Cancer. 2020 Jan 24;19:15. doi: 10.1186/s12943-020-1141-9 (PMC6982393; doi:10.1186/s12943-020-1141-9)
Supplement: Supplementary file 2 — Additional file 2 : Table S2. Current studies on the prognostic value of CTCs in immunotherapeutic strategies. Table S3. PD-L1 status in CTCs of patients before and after the initiation of IBI308 therapy. [file 12943_2020_1141_MOESM2_ESM.docx]

**Table S2.** Current studies on the prognostic value of CTCs in immunotherapeutic strategies

| Immunotherapeutic  Strategies | Cancer type | Observation index | Index analysis | References |
| --- | --- | --- | --- | --- |
| NK cell therapy | NSCLC | Number of CTCs | The number of CTCs decreased significantly at 7 and 30 d after NK cell therapy (P < 0.01)，which suggested that enumeration of CTCs can be used to evaluate the efficacy of NK cell therapy. | [1] |
| NK cell therapy | Hepatic carcinoma | Number of CTCs | The decrease in the number of CTCs may have been related to tumor shrinkage and have reflected the efficacy of NK cell therapy. | [2] |
| Combination therapy of IRE and NK cell immunotherapy | Unresectable primary liver cancer | Number of CTCs | The combination therapy of IRE and NK cell immunotherapy significantly reduced CTCs, and therefore, the decrease in CTCs may reflect the improved efficacy of the combination therapy. | [3] |
| Nivolumab | NSCLC | Number of CTCs and PD-L1 positive CTCs | The presence of pretreatment PD-L1+ CTCs was not significantly correlated with outcomes, but pretreatment PD-L1+ CTCs were associated with a bad prognosis in patients treated with PD-1 inhibitors. | [4] |
| Nivolumab | NSCLC | Number of CTCs and PD-L1 positive CTCs | The presence of CTCs and the expression of PD-L1 on their surface were found associated to poor patients’ outcome, Patients with PD-L1 negative CTCs all obtained a clinical benefit, while patients with PD-L1 (+) CTCs all experienced progressive disease. | [5] |
| Nivolumab | HNC | Number of CTCs and PD-L1 positive CTCs | CTC‐positive patients had a shorter PFS, and PD‐L1‐positive CTCs were significantly associated with worse outcomes. | [6] |
| IBI308 | Nultiple cancer ***(Table.S3)*** | PD-L1 expression levels on CTCs with four categories (PD-L1negative, PD-L1low, PD-L1medium and PD-L1high) | Patients with PD-L1-overexpressing tumors achieved improved clinical outcomes during PD-1/PD-L1 blockade therapy. The number of PD-L1-high CTCs was associated with prognosis. | [7] |
| Whole-cell melanoma vaccine plus Bacille-Calmette-Guerin (BCG) | Nelanoma | MART-1, MAGE-A3 and PAX3mRNA expression level | DFS was significantly worse for patients with >0 vs. 0 positive biomarkers. | [8] |

**Table S3** PD-L1 status in CTCs of patients before and after the initiation of IBI308 therapy [7]

References

1. Lin M, Liang SZ, Shi J, Niu LZ, Chen JB, Zhang MJ, et al. Circulating tumor cell as a biomarker for evaluating allogenic NK cell immunotherapy on stage IV non-small cell lung cancer. Immunol Lett. 2017; 191:10-5.

2. Qin Z, Chen J, Zeng J, Niu L, Xie S, Wang X, et al. Effect of NK cell immunotherapy on immune function in patients with hepatic carcinoma: A preliminary clinical study. Cancer Biol Ther. 2017; 18(5):323-30.

3. Yang Y, Qin Z, Du D, Wu Y, Qiu S, Mu F, et al. Safety and Short-Term Efficacy of Irreversible Electroporation and Allogenic Natural Killer Cell Immunotherapy Combination in the Treatment of Patients with Unresectable Primary Liver Cancer. Cardiovasc Intervent Radiol. 2019; 42(1):48-59.

4. Guibert N, Delaunay M, Lusque A, Boubekeur N, Rouquette I, Clermont E, et al. PD-L1 expression in circulating tumor cells of advanced non-small cell lung cancer patients treated with nivolumab. Lung Cancer. 2018; 120:108-12.

5. Nicolazzo C, Raimondi C, Mancini M, Caponnetto S, Gradilone A, Gandini O, et al. Monitoring PD-L1 positive circulating tumor cells in non-small cell lung cancer patients treated with the PD-1 inhibitor Nivolumab. Sci Rep. 2016; 6:31726.

6. Kulasinghe A, Kapeleris J, Kimberley R, Mattarollo SR, Thompson EW, Thiery JP, et al. The prognostic significance of circulating tumor cells in head and neck and non-small-cell lung cancer. Cancer Med. 2018; 7(12):5910-9.

7. Yue C, Jiang Y, Li P, Wang Y, Xue J, Li N, et al. Dynamic change of PD-L1 expression on circulating tumor cells in advanced solid tumor patients undergoing PD-1 blockade therapy. Oncoimmunology. 2018; 7(7):e1438111.

8. Hoshimoto S, Faries MB, Morton DL, Shingai T, Kuo C, Wang HJ, et al. Assessment of prognostic circulating tumor cells in a phase III trial of adjuvant immunotherapy after complete resection of stage IV melanoma. Ann Surg. 2012; 255(2):357-62.
